# Creation of Rice Doubled Haploids Resistant to Prolonged Flooding Using Anther Culture

**DOI:** 10.3390/plants12213681

**Published:** 2023-10-25

**Authors:** Pavel Kostylev, Nataliya Kalinina, Nataliya Vozhzhova, Valentina Golubova, Natalya Chertkova

**Affiliations:** FSBSI Agricultural Research Center “Donskoy”, Nauchny Gorodok, 3, 347740 Zernograd, Rostov Region, Russia; kalinina74783@mail.ru (N.K.); nvozhzh@gmail.com (N.V.); valya_l7@rambler.ru (V.G.); tycik17082012@gmail.com (N.C.)

**Keywords:** rice, grade, hybrid, lines, anther, induction, callus, regeneration, dihaploid, flood resistance

## Abstract

Flood resistance in rice is very important in weed control, as weeds cannot overcome deep water. At present, there are no released varieties in Russia that would meet these requirements. The creation of such varieties will reduce production costs and pesticide load on the ecosystem. The object of the study was second-generation rice hybrids obtained by crossing the best varieties for economically valuable traits with samples carrying genes for resistance to prolonged flooding with water. To create double rice haploids resistant to prolonged flooding, the anther culture method was used, followed by molecular genetic evaluation of dihaploids for the presence of genes for resistance to prolonged flooding. An estimate of the growth energy under deep flooding was carried out according to our own method. As a result of the cultivation of anthers, 130 androgenic regenerated plants were obtained in 14 hybrid combinations. In terms of responsiveness to neoplasms, 60% of the panicles showed a positive result, while the rest 40% did not demonstrate callus formation. In total, 30 green regenerative lines were obtained from four rice hybrids, differing in visual morphological assessment. Large genotypic differences between the samples were revealed. These lines carry long-term flood resistance genes and can be used in rice breeding programs using dihaploids. As a result of the assessment of the growth energy in a number of obtained samples, the potential for rapid elongation of the first leaves, overcoming a large layer of water and accumulation of vegetative mass, was revealed.

## 1. Introduction

Rice is one of the main food sources for more than half of the world’s population and is the third largest grain producer. Breeders are constantly looking for biotic and abiotic stress resistance mechanisms to improve yields under stressful conditions. Great importance is attached to increasing flood resistance. If flooding persists for several days, then crops of unstable varieties die, and yields are lost [1,2].

During vegetative growth, rice uses various flood protection strategies. Genetic variability of plant response includes various schemes: (1) dormancy, which allows to withstand a long time under water, (2) a strategy of rapid stem elongation with changes in plant structure and metabolism [1].

The SUB1A locus belongs to the genes that control flood resistance, which manifests itself in waiting for the onset of an unfavorable factor. The dormant state is observed in rice varieties with the SUB1A locus, which limit starch mobilization and thus produce less ethanol and other fermentation products, and also affect the processes associated with aging [1,3].

The discovery of the main quantitative trait loci Sub1 (QTL) on rice chromosome 9, the principal contributor to flooding tolerance, was a significant step forward in flooding tolerance research [3].

Researchers have identified two additional loci associated with flood resistance: SNORKEL 1 and SNORKEL 2 (chromosome 12). Plants with these genes show rapid growth and coleoptile elongation during development under anaerobic conditions, which is called the “tube effect” [4].

This type of adaptation mechanism prevails in deep-sea lowland rice, where a significant elongation of shoots keeps the plant from completely submerging with a constant increase in flood water levels. SK1 and SK2, genes of ethylene sensitivity factors, which were found in the deep-sea rice variety C9285, play a vital role in lengthening the internodes of plant shoots in response to immersion. Both genes act in opposition to Sub1. The Sub1a gene allows the rice plant to withstand flash floods by limiting the elongation of shoots until flood waters recede. The slower lengthening of coleoptiles, the development of tolerant varieties adapted to the conditions of sudden floods, thus preserves the accumulated biomass, which is used to continue growth after leaving the water [4].

An important aspect of the use of rice flood resistance, most relevant for Russia, is the fight against weeds due to the deep water layer, which weeds cannot overcome and die. This leads to positive effects such as the absence of the need to use herbicides, a reduction in the cost of a unit of production, an increase in the quality of products that can be used in the production of products for children and diet food, and the absence of environmental damage [5,6].

Varieties with genes for this resistance have been found in Asia. It is necessary to transfer these genes into the germplasm of Russian varieties. To speed up this process, it is necessary to rationally combine classical breeding methods with biotechnological methods, which allows solving the tasks set in a shorter time [7]. Therefore, in addition to the classical methods of creating new rice varieties, androgenesis and haploidy are widely used [8]. Rice growing countries of the world have long used the cultivation of anthers on an artificial nutrient medium in breeding work [9,10,11]. The use of anther culture in breeding work allows for quickly obtaining homozygous plants that are resistant to various stressful environmental conditions, including drought, soil salinity, extreme temperatures and diseases.

Growing anthers on an artificial nutrient medium makes it possible to obtain rice haploids and homozygous dihaploids in 1–2 years. Extensive work on this technique is being carried out at the Federal State Budget Scientific Institution “Federal Science Center of Rice” [12,13,14] and the Primorsky Research Institute [15,16]. In order to obtain hundreds and thousands of lines in anther culture in vitro, it is necessary to ensure a large percentage of regeneration of green buds and plants in each hybrid combination [17,18,19]. Indicators of regenerative ability vary greatly depending on the hybrid and genotype, i.e., even within the same hybrid combination; different plants provide different rates of callus formation and regeneration of green plants from very low to very high. Thus, it is necessary to cover as many hybrid plant genotypes as possible for use in in vitro culture [20,21,22].

The aim of the study was to obtain rice dihaploids by cultivating anthers in vitro to accelerate selection for resistance to prolonged flooding.

## 2. Materials and Methods

Donor plants and growth conditions. The object of the study was rice hybrids of the second generation, obtained by crossing the best varieties for economically valuable traits with samples that do not have genes for resistance to prolonged flooding (Table 1).

The flood resistance locus SUB1A was transferred from donors Inbara-3, IR-64, Br-11, CR-1009, TDK-1. The donors of the SNORKEL 1, 2 genes were Khao Hlan On, Kharsu 80A, and Ma-Zhan Red. Local varieties Novator, Magnat, Contact and Kuboyar were used as a control.

Panicle selection and pre-treatment. The selection of panicles was carried out in the fields of the laboratory of rice breeding and seed production of the Agricultural Research Center “Donskoy” in Proletarsk, Rostov Region, in 2022 in the morning, in clear weather.

A morphological trait suitable for the selection of shoots was the distance from the ear of the flag leaf to the ear of the next leaf, which should be from 5 to 10 cm [4,6,23].

Selected shoots were subjected to surface sterilization with 96% alcohol for 3 min. After that, they were placed in vessels with water, covered with a plastic bag, and exposed to low positive temperatures (5 °C) for 7–10 days in a refrigerator (Liebherr-Hausgeraete, Ochsenhausen, Germany).

Panicle sterilization. Before introduction into the culture, flag leaves were removed from the panicles, twigs with pollen grains in the stage of medium and late mononuclear microspores were selected according to morphological characteristics and the rest were removed. The twigs were wrapped in sterile gauze and loosely fastened with thread. Then, they were immersed in a sterilizing solution of 5% sodium hypochlorite (Sigma, St. Louis, MI. USA) for 10 min and washed three times in sterile distilled water.

**Figure 1:**
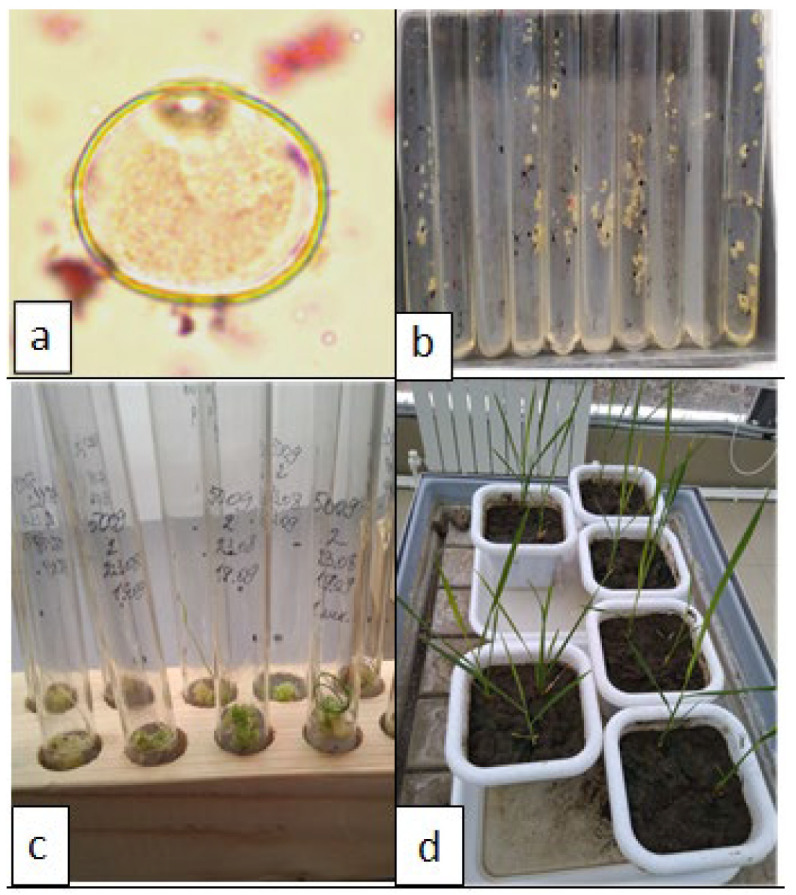
Stages of obtaining rice regenerant plants in anther culture in vitro: (**a**)—microspores in the middle single-core stage; (**b**)—neoplasms on anthers; (**c**)—morphogenesis on calluses; (**d**)—cultivation of regenerating plants in the soil.

Determination of the stage of development of microspores. From each panicle, from its middle part, 2–3 spikelets were isolated to determine the stage of development of microspores. Spikelets were placed on a glass slide and anthers were removed using dissecting needles. Then, the anther was cut across and the microspores were squeezed out, while two drops of acetocarmine (Merck Peruana S.A., Lima, Perú) were added, heated over an alcohol burner and left for staining (10–15 min). After that, the preparation was covered with a cover slip and examined under a microscope (ADF U300FL, Ningbo, China) (see Figure 1a). The most appropriate stage in the development of microspores is the transition from the late single-core to the early two-core stage. However, the middle uninuclear stage of microspore development has been identified as optimal for an effective androgenic response [1].

Preparation medium. Callus formation was induced from rice anthers on Blades nutrient medium containing 2.0 mg/L 2,4-D (Sigma, USA), 30 g/L sucrose (Panreac, Barcelona, Spain) and 8 g/L agar (MerckRGaA, Saint Louis, USA). For regeneration, morphogenic callus was transplanted onto Murashige and Skoog (MS) base medium with 1.0 mg/L NAA (Sigma, USA), 5.0 mg/L kinetin (Sigma, USA), 20 g/L sucrose, and 8 g/L agar (Table 2). The prepared nutrient medium was poured into test tubes with a diameter of 20 mm, autoclaved (Tuttnauer 3870 ELV, Beit Shemesh, Israel) for 15 min at a temperature of 121 °C and a pressure of 0.9–1.0 atm. Immediately after sterilization, tubes with hot medium were placed at an angle of about 30 °C to obtain an inclined agar and left to solidify [7,14].

Anther inoculation and callus induction. Anthers were inoculated under aseptic conditions in a laminar flow hood (BMB-II-Laminar-S, Miass, Russia), which was sterilized with ultraviolet lamps. The instruments used were also partially sterilized (SteriMaxsmart-WLD-TEC, Arenshausen, Germany). All items needed in the working process were treated with 96% ethyl alcohol and then burned in the flame of an alcohol burner. Sterile instruments were placed between sheets of thick wrapping paper previously sterilized by dry heat in an oven (BinderFD23, Tuttlingen, Germany) at 130 °C for 2 h (from the moment the set temperature was set).

Anthers were separated from the spikelets and transferred to the medium in an amount of up to 30 pcs. into a test tube using a scalpel, tweezers and a dissecting needle, closed it with a foil cap and placed in a thermostat with a temperature of 28 ± 2 °C and a relative humidity of about 50% (Binder KV400, Tuttlingen, Germany) for 30–50 days (Figure 1b).

Planting calluses and plant regeneration. The morphology of calli is closely related to their ability to regenerate plants [14]. The morphological assessment of rice calluses can be described as follows:

(1) With meristematic foci, light shades, fine-grained, medium density (morphogenic);
(2) Spherical, white, light yellow, medium density (morphogenic);
(3) Dense, white, fine-grained (morphogenic);
(4) Loose, moist, with vascular cords (rhizogene);
(5) Granular, loose, brown, with large cells (very low ability to morphogenesis);
(6) Watery, dark brown, with large shapeless cells of different sizes (non-morphogenic).

Morphogenic calli 1 mm in size or more were placed on Murashige and Skoog regeneration medium poured into test tubes 20 mm in diameter (Figure 1c). Tubes with callus explants were incubated in an illuminated growth room with a temperature control of 25 ± 2 °C, illumination of 2000 Lux, and a photoperiod of 15 h/9 h. After 15–20 days, regenerated plants were formed in the light [6,7].

Acclimatization of regenerative plants and planting them in the soil. Regenerants with a developed root system and 4–5 leaves at least 8 cm long were planted in a pot culture (Figure 1d). The root system was thoroughly washed from the agar medium and left for a day in glasses with water. The soil was preliminarily sieved, sterilized (3 h in a dry oven at a temperature of +110 °C) and moistened. Containers with plants were placed in a bright room, without direct lighting, so that a sharp change in environmental conditions would not have a negative effect on the plants.

After that, the regenerator plants were transferred to the greenhouse, where their development continued until flowering, seed formation and maturation. The following microclimate parameters were favorable for rice plants: daytime temperature 25 °C, illumination more than 5000 lux; night temperature 20 °C, illumination 0 lux; humidity 70–80%; photoperiod 12 h [6,7].

One feeding session was carried out with a nutrient solution (1/2 MS). Plants were watered 3–4 times a week, depending on the drying of the soil in the growing vessels.

Diagnosis of plant ploidy. To restore fertility in haploid plants, it is necessary to induce chromosome duplication. However, rice haploids obtained by cultivating anthers usually undergo spontaneous diploidization. In this work, plants were not treated with polyploidizing agents.

The ploidy of the resulting regenerative plants at the stage of growth and development (before seed formation) was determined by morphological traits such as plant height and leaf size. Plants with normal morphological features were considered doubled haploids. Haploid plants [23] had a low plant height and narrow leaves. The tetraploids were very tall, with large leaves. Simultaneously, the content of nuclear DNA in different groups of regenerated plants was determined. The DNA content was determined using a flow cytometer (MuseCellAnalyzer, Austin, TX, USA). Green regenerative plants obtained from calli were evaluated. The plant material (leaves) was subjected to freeze-drying.

A leaf sample with an area of 1–2 cm^2^ was crushed with a spatula in a Petri dish in 1 mL of cooled Tris-MgCl_2_ buffer. The buffer contained 0.2 m Tris base (Molekula Ltd., Darlington, UK), 4 mM MgCl_2_ × 6H_2_O (Amresco, Solon, OH, USA) and 0.5% TritonX-100 (Sigma, USA) supplemented with 1 µL/mL β-mercaptoethanol (Sigma, St. Louis, MI, USA), 50 µg/mL propidium iodide (Sigma, USA) and 50 µg/mL RNase (Syntol, Moscow, Russia). The samples were filtered through a nylon membrane filter with a pore size of 50 μm. *Ficus benjamin* L. embryos isolated in a similar way with a known DNA content of 2C = 1.07 pg were used as an external standard [24,25].

Molecular genetic assessment of the presence of flood resistance genes.

All obtained regenerative rice lines were evaluated by two molecular markers (Table 3).

The genomic DNA of the isolated samples was extracted from freshly cut leaves by the CTAB method (Panreac, Barcelona, Spain) on a homogenizer (Bertin Precellys 24, Montigny le Bretonneux, France). Homogenization was carried out in two stages of 30 s, at a speed of 3000 rpm. The quantity and quality of the isolated DNA were assessed using a spectrophotometer (Implen Nanophotometr NP80, Munich, Germany). The isolated DNA was placed in an amplifier (Bio-RadT100, Richmond, CA, USA) for PCR. The samples were then placed in a gel electrophoresis chamber. The gel was prepared on the basis of a 0.5-fold TBE buffer 10 × (Evrogen, Moscow, Russia) (100 mL) and agarose (Amresco, Solon, OH, USA) (2 g). After PCR, 3–5 µL of 6× TriK dye (Biolabmix, Novosibirsk, Russia) was added to each tube with a sample after PCR. A molecular weight marker was also applied to the agarose gel. The duration of electrophoresis ranges from 30 min to 2 h, depending on the expected size of the amplificates. Then, the gel plates were placed for 20–30 min in a solution of ethidium bromide fluorescent dye (Sigma, USA) for staining. After that, the gel was removed, carefully washed in a cuvette, and photographed under ultraviolet light using a gel photodocumentation system (Bio-RadGelDocXR+, Richmond, VA, USA) [26].

**Figure 2:**
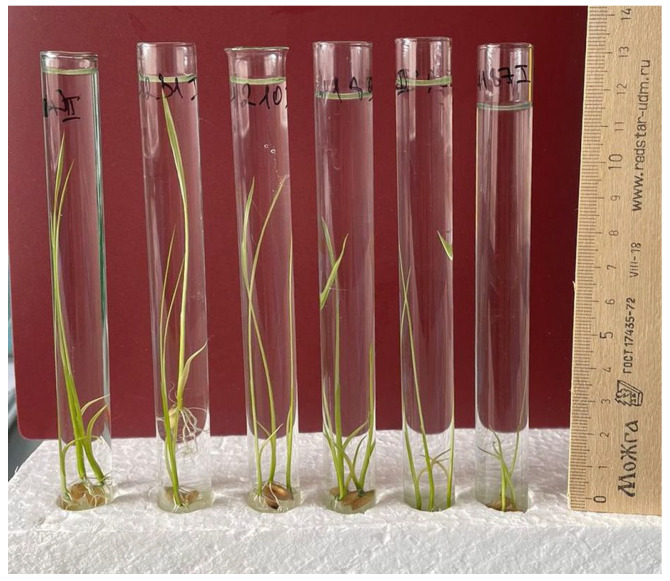
Germination of rice seeds in test tubes under stress.

Evaluation of resistance of rice samples to long-term flooding by the method of germination under stress conditions. To estimate the growth energy during prolonged flooding, our own method was developed. Glass test tubes (1.5 cm in diameter and 15 cm in height) were used for rice seed germination and plant growth. The seeds were placed in a test tube, filled with distilled water to a depth of 10 cm, and incubated in the light under room conditions at a temperature of 28 °C (Figure 2).

**Table 3:** Molecular markers of quantitative trait loci associated with long-term flood resistance in rice.

Gene	Primer	Subsequence (5′–3′)	Size (bp)	Link
SUB1A	RM 7481 F	CGA CCC AAT ATC TTT CTG CC	95	Azarin et al., 2016 [27]
	RM 7481 R	ATT GGT CGT GCT CAA CAA G		
SNORKEL1	1F	ATG TGC GGA GGT TGT CTC AT	743	Oe et al., 2021 [28]
	1R	TCG TAG CGA CAG CCG TAC TG		

On the 5th, 7th, 9th, 11th days, the length of the sprout was measured to determine the growth dynamics.

Observations and statistical analysis. Mathematical and statistical data processing was carried out in MS Excel.

## 3. Results and Discussion

Hybrids of Russian rice varieties with flood resistance donors were obtained in 2012–2013. The donors of the Sub1A gene were crossed with the varieties Novator, Contakt, Magnat, and the donors of the Snorkel and AG genes were crossed with the varieties Contakt and Kuboyar. The donors were extremely late-ripening and photosensitive; therefore, in subsequent years, mid-ripening recombinants capable of maturing in the Rostov region were selected. As a result of long-term selection of forms with a complex of economically valuable traits, a group of self-pollinated lines was formed, among which there were plants with resistance genes. To identify them, physiological and genetic studies were carried out, during which lines with genes of interest were selected. Subsequently, they were crossed with the best varieties and among themselves for the pyramiding of genes.

To speed up the selection process, it is necessary to obtain homozygous lines from second-generation hybrids using androgenesis.

The creation of double rice haploids resistant to prolonged flooding by the anther culture method is a two-stage process: the initial development of the callus and the subsequent regeneration of green plants from the embryogenic callus [13,25]. Features of androgenesis in hybrid combinations of rice were studied by cultivating anthers on Blades induction medium.

As a result of the experiment, in 2022, 12,604 anthers were extracted from 68 hybrid rice panicles (26 crossing combinations) and planted on an induction nutrient medium. The maximum number of anthers was planted in hybrid 5016/2—339 pieces, and the minimum was 4773/1—47 pieces.

When anthers of all genotypes were cultivated on an induction medium, neoplasms appeared from them—embryo-like structures (single embryoids and polyembryoids) and callus. When cultivated on a regenerative medium, as a rule, the callus and part of the embryonic structures remained unchanged. Some of the germ-like structures developed roots, while another part had solitary seedlings or clusters of seedlings. The appearance of callus and germ-like structures began on the 30–33rd day from the moment the anthers were planted on the nutrient medium. Dense or slightly transparent, well-defined neoplasms were classified as embryo-like structures. Callus formation continued for another four weeks; the anthers were on the medium for two months.

As follows from the above data, not all hybrid combinations of rice showed the ability to form callus and embryo-like structures. The amount of callus formation in the anther culture varied significantly both between hybrid combinations and in different plants from the same hybrid combination, which, apparently, is due to genotypic differences, as well as the influence of external factors (explant quality, growing conditions etc.). In total, 716 neoplasms were obtained, which is, on average, 10 pcs. per plant, taking into account non-susceptible ones (Table 4).

According to the ability to neoplasms, 60% of the volume of the studied material (39 panicles) showed a positive result, while 40% (29 pcs.) did not demonstrate callus formation. The most sensitive to callus formation were the following four hybrid combinations: 5009/2—84 pcs., 5010/2—94 pcs., 4565/3—85 pcs., 4641/2—69 pcs. (Table 2). The same samples showed the ability to carry out morphogenesis, while the remaining combinations formed a non-morphogenic callus (in some cases up to 100%). Samples 5007, 5006, 5011 and 4585 showed no response to in vitro culture.

It was found that not only hybrid combinations differed among themselves in the their callus formation abilities, but also individual plants within the same combination. For example, in sample 5103, anthers were planted from three plants on a nutrient medium with 259, 245, and 110 pieces. Of these, 20, 2 and 0 calli were formed, respectively, but only 4 regenerated plants appeared from the first one. From sample 4641, an equal number of anthers was taken from two plants, including 193 and 194 pieces. However, in the first one, 5 calli were found, and in the second, 69 were found, that is, 14 times more. The former subsequently developed only 1 regenerating plant, which was albino, while the latter had 18 green plants and 3 without chlorophyll. This indicates the presence of genetic factors that significantly affect the ability of cells to callus formation and regeneration.

Green seedlings and albinos developed from the formed neoplasms during cultivation on a regenerative medium; structures developing by the type of root formation and structures without development were also noted.

The ability of calli to carry out morphogenesis was assessed by plant regeneration. The studied rice samples formed 130 regenerated plants from 14 hybrid combinations. Of these, only 25 plants were green. In total, four samples were isolated that formed regenerants without chlorophyll defects in the leaves—5009/2 [(Inbara 3 × Novator) × Contact] − 3 pcs., 5010/2 [(Inbara 3 × Novator) × Contact] − 6 pcs., 4565/3 (IR64 × Magnate) − 2 pcs., 4641/2 [(Inbara-3 × Contact) × (Khao Hlan On × Kuboyar)] − 14 pcs. Albino plants died at the early stages of development, as they were not capable of photosynthesis and, accordingly, of the autotrophic type of nutrition. The largest number of albinos was formed in the sample 5021/1—25 pcs.

As a result of evaluating the efficiency of anther cultivation in rice hybrids, it was found that the largest number of neoplasms per 100 cultivated anthers (76.9) was obtained in sample 5009/2, which significantly exceeded the average value in the experiment (Table 5).

Morphogenesis and the ability of neoplasms to form seedlings, including green and albinos, were assessed on the basis of the indicator “the number of all regenerants per 100 planted anthers.” More plants were regenerated based on the hybrid combination 5009/2, which was significantly higher than the average value. In general, more regenerants were formed in samples 5009/2 and 4641/2: 14.3 and 10.8, respectively.

Green plants are of practical interest; therefore, the most important indicator of anther culture is the sign “number of green regenerants per 100 isolated anthers”. According to this characteristic, sample 4641/2 had a higher value (9.3). The indicators of other genotypes were at the level of the average value.

To assess the ability of neoplasms to regenerate seedlings, the number of all regenerants per 100 neoplasms was determined. The maximum value of this characteristic was in the hybrid combination 4641/2 (30.4). On average, new growths of sample 5009/2 (20.2) also regenerated plants well. The remaining combinations formed fewer plants, but within the average of the experiment.

Accession 4641 was obtained as a result of stepwise hybridization of the Inbara-3 variety (Sub1A gene), which is resistant to deep flooding, with the Russian early maturing variety Contact, and then the fifth-generation line was used when crossing with a vigorously growing accession (Khao Hlan On × Kubojar) carrying the Sk1 gene. These genes allow seeds to germinate quickly and plants to vigorously overcome the water layer. Apparently, this ability also affected the regenerative ability of callus cells, dramatically increasing the number of regenerative plants.

It was established that regenerated plants were obtained from combinations with long-term flood resistance genes: (Inbara-3 × Novator) × Contact (5009/2; 5010/2), IR-64 × Magnat (4565/3)—Sub1A gene, from the combination (Inbara-3 × Contact) × (Khao Hlan On x Kubojar) (4641)—Sk1 gene.

Following from the results obtained in this work, there are strong differences in the manifestation of signs of androgenesis in the anther culture between second-generation rice hybrids carrying the target Sub1A, Snorkel 1, 2 genes. At the same time, the low ability for androgenesis in these hybrids could depend on many factors. It has been established that the composition of the nutrient medium for growing rice anthers most closely matches the type of nutrition of this crop. There are studies showing that the rate and frequency of callus induction can be increased up to 27.9%, and even higher in some types of genes [6,29]. These data are consistent with the studies conducted in our work, namely, the inclusion of 2,4-D in the induction medium of 2.0 mg/L made it possible to obtain up to 70% of neoplasms per 100 cultivated anthers.

In the course of the work, the quantity and quality of calli were taken into account for each hybrid combination. Morphogenic calli were light, opaque, compact and had green areas containing chlorophyll, which were zones of morphogenesis. This is confirmed in the works of other authors [30].

The activation of morphogenesis processes and plant regeneration is stimulated by the use of 1.0 mg/L of alpha-naphthylacetic acid (NAA) in combination with 5.0 mg/L of kinetin. For example, in some works, samples with a low regenerative capacity (0.31–0.72%) were isolated, in addition to medium (6.07–7.61%) and high (21.65–27.9%) [7,28]. The results are also confirmed in our studies. Among the selected hybrid combinations of rice, the number of all regenerants per 100 neoplasms reached a maximum value of 30%, including green ones—from 1 to 9%, i.e., samples showed low and medium regenerative capacities.

In most rice plants obtained by cultivating anthers, spontaneous duplication of chromosomes occurs [26]. This is confirmed in our studies (Table 6). The obtained green regenerated plants were divided into three groups depending on the content of nuclear DNA: haploids, diploids and tetraploids.

All plants within their group were characterized by insignificant variability in the content of DNA in cell nuclei. Our data are comparable with the results of other authors, according to which the content of nuclear DNA in the main set of chromosomes in *O. sativa* rice ranges from 0.91 to 1.00 pg [24,26]. The ratio of the average values of the content of nuclear DNA in dihaploids and haploids was not a multiple of two. This may indirectly indicate the loss of some parts of the chromosomes in haploids during cultivation, which leads to changes in the morphotype of the regenerants.

Assessing the level of ploidy in regenerants is the most important key step in applying androgenesis to a breeding program. In our study, morphological evaluation was found to be sufficiently reliable to distinguish diploids from other ploidy levels and was performed quickly and easily. Although flow cytometry is an attractive approach for assessing the ploidy level of regenerated plants, its use is still limited in many laboratories due to the high cost of equipment and higher analysis costs [29,31,32]. The flow cytometry method in combination with morphological assessment can be used in rice breeding to identify the ploidy of regenerants, as well as to cull haploids in order to exclude the stage of growing unpromising forms under ex vitro conditions.

Regenerative anther-derived rice lines surviving in pot culture under greenhouse conditions were assessed using selected molecular markers identifying alleles of the Sub1A, Sk1, 2, and AG genes.

**Figure 3:**
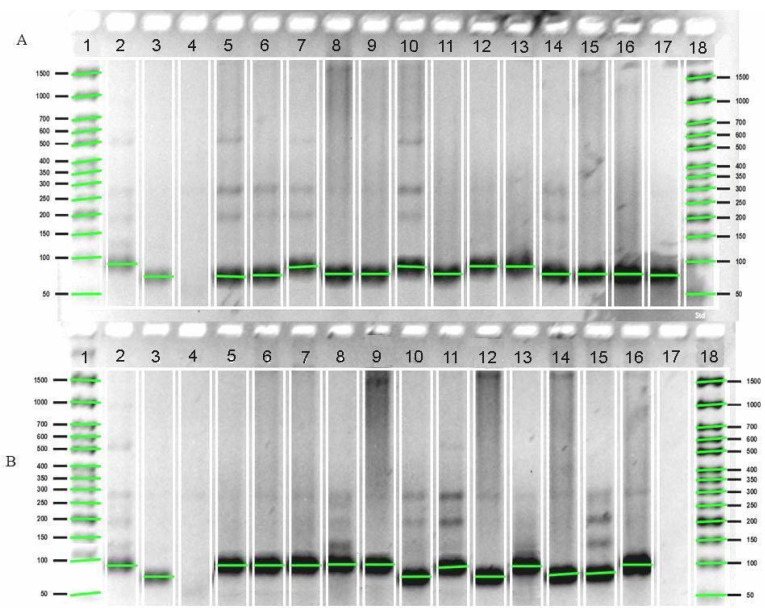
Electrophoregram of screening rice regenerative plants for the presence of the SUB1A gene with a molecular marker RM7481. (**A**) Line 1—molecular weight marker Biolabmix Step 50+ (50–1500 bp), 2—Inbara 3 (Sub 1A donor), 3—Novator (recessive), 4—deionized water (internal experience control), 5—4641/1, 6—5010/4, 7—5010/3, 8—4641/2, 9—4641/14, 10—4641/13, 11—4565/1, 12—4565/2, 13—4641/7, 14—5009/1, 15—4641/3, 16—5009/3, 17—5010/6, 18—Molecular weight marker Biolabmix Step 50+ (50–1500 bp). (**B**) Line 1—molecular weight marker Biolabmix Step 50+ (50–1500 bp), 2—Inbara 3 (Sub 1 donor), 3—Novator (recessive), 4—deionized H_2_O (internal control experience), 5—4641/10, 6—4641/12, 7—4641/8, 8—4641/5, 9—5010/2, 10—4641/9, 11—5010/5, 12—4641/6, 13—5010/1, 14—4641/11, 15—4641/4, 16—5009/2, 17—deionized water, 18—molecular weight marker Biolabmix Step 50+ (50–1500 bp).

The Sub1 locus is considered to be the main gene controlling flood resistance. As a result of molecular genetic analyzes conducted in 2023, the presence of Sub1A long-term flood resistance genes was found in rice regenerants. Electrophoregrams of sample identification for the Sub1A gene are shown in Figure 3.

In Figure 3A, samples 5010/3, 4641/13, 4565/2, 4641/7 (lines 7, 10, 12, 13) corresponds to the Inbara 3 control (line 2), while in Figure 3B, samples 4641/10, 4641/12, 4641/8, 4641/5, 5010/2, 5010/1, 5009/2 (lines 5–9, 11, 13 and 16) correspond to the Inbara 3 control (line 2). These accessions carry the Sub1A long-term flood resistance gene. The Sub1A locus belongs to the genes that control resistance to flooding, which manifests itself in waiting for the impact of an unfavorable factor.

When a submerged coleoptile reaches the surface of the water, the hollow structure of the coleoptile allows O_2_ to escape from the surface to the submerged parts of the plant. Thus, elongation of coleoptiles is a strategy of the rice plant to avoid stress during prolonged flooding, and is called the “tube effect” [33]. Plants with Snorkel 1, 2 (Sk 1, 2) genes show rapid growth and coleoptile elongation during development under anaerobic conditions.

**Figure 4:**
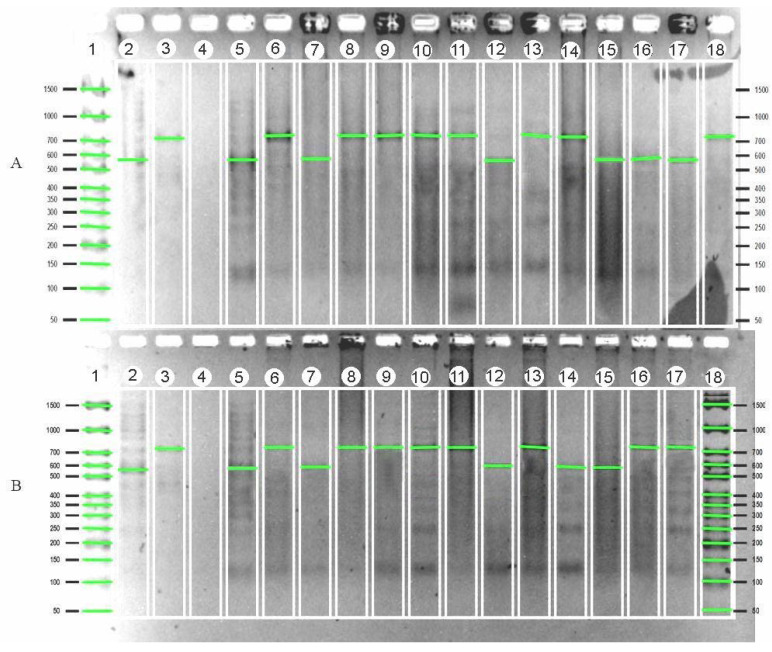
Electrophoregram of screening rice regenerative plants for the presence of the gene Sk1 with molecular marker F1R1. (**A**) Line 1—Molecular weight marker Biolambix Step 50+ (50–1500 bp), 2—Bahus (vigorously growing), 3—Khao Hlan On (vigorously growing), 4—deionized H_2_O (internal control experience), 5—Boyarin (slow growing), 6—4641/14, 7—4641/2, 8—4641/1, 9—4641/13, 10—5009/2, 11—4641/5, 12—4641/7, 13—5009/1, 14—4641/8, 15—5009/3, 16—5010/6, 17—4641/10, 18—5010/2. (**B**) Line 1—Molecular weight marker Biolambix Step 50+ (50–1500 bp), 2—Bahus (vigorously growing), 3—Khao Hlan On (vigorously growing), 4—deionized H_2_O (internal control experience), 5—Boyarin (slow growing), 6—4641/3, 7—4565/2, 8—4641/12, 9—4641/9, 10—5010/5, 11—4641/6, 12—5010/1, 13—4641/11, 14—4641/4, 15—4565/1, 16—5010/3, 17—5010/4, 18—Marker (Biolabmix Step 50+).

In Figure 4A, the samples (line 6, 8–11, 13, 14, 18) correspond to the control Khao Hlan On (line 3) and samples (lines 5, 7, 12, 15–17) correspond to the Bakhus control (line 2). In Figure 4B, the samples (lines 6, 8–11, 13, 16, 17) correspond to the control Khao Hlan On (line 3). The isolated accessions carry the Sk1 long-term flood resistance gene.

**Figure 5:**
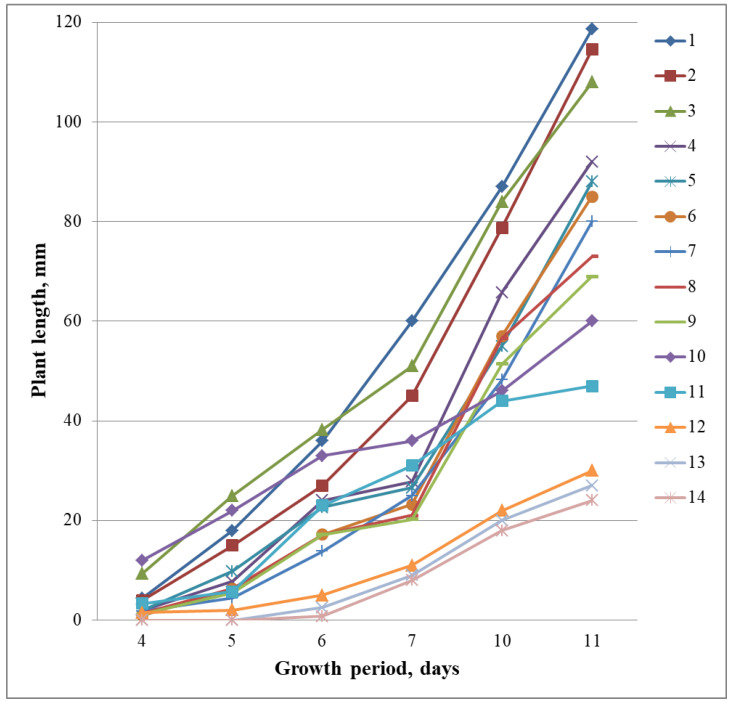
Growth dynamics of rice plants in test tubes under water. Regenerated lines: 1—4641/12; 2—4641/3; 3—4641/1; 4—4641/11; 5—4641/8; 6—4641/10; 7—4641/2; 8—4641/4; 9—4641/14; 10—5010/6; 11—4641/9; 12—4641/7; 13—4641/5; 14—5009/2.

The results of the studies showed significant differences between plants that are descendants of regenerants in terms of their growth rate under water in test tubes (Figure 5). After 5 days from the beginning of germination, the average length of sprouts varied from 0 to 25 mm. On the 7th day, the differences between the samples became more significant, from 8 to 60 mm. On the 11th day of growth, plant height varied from 24 to 119 mm (Figure 5).

By the 11th day, the maximum length of plants was more than 108–118 mm in samples with a locus in the genotype Sk1: 1—4641/12, 2—4641/3 and 3—4641/1. They rose above the layer of water and the top edge of the tube. Samples with the gene Sub1A: 12—4641/7; 13—4641/5; 14—5009/2 with a stem length 24–30 mm were noted.

Thus, as a result of the physiological evaluation of rice samples, vigorously growing homozygous lines with the greatest potential for growth and development were identified. Of interest are also samples that inhibit their growth to save nutrients, which is due to the Sub1A gene.

**Figure 6:**
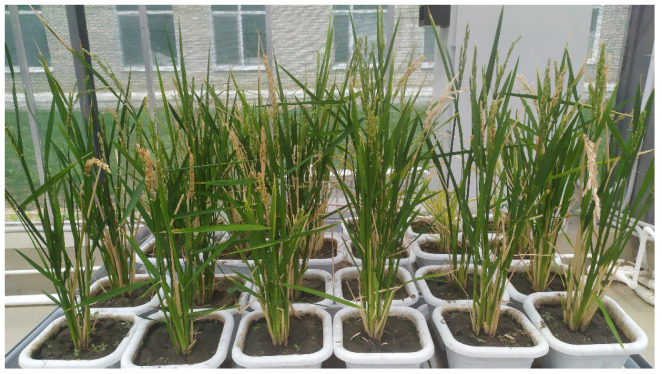
Appearance of rice regenerating plants, 2023 Zernograd.

Regenerant plants were planted in a greenhouse in lysimeters, where they grew to adulthood (Figure 6). All of them have expanded, forming from 3 to 20 panicles per plant. The height of the plants was slightly lower than in the field, and varied from 26 to 74 cm. The length of the panicle ranged from 4 to 17 cm. The number of spikelets on the panicle ranged from 40 to 128 pieces. At the same time, high sterility was observed. The number of grains on the panicles of diploid forms ranged from 18 to 104 pieces. Tetraploid plants formed from two to four grains (Table 7). Haploid forms were completely sterile and did not form grain.

**Figure 7:**
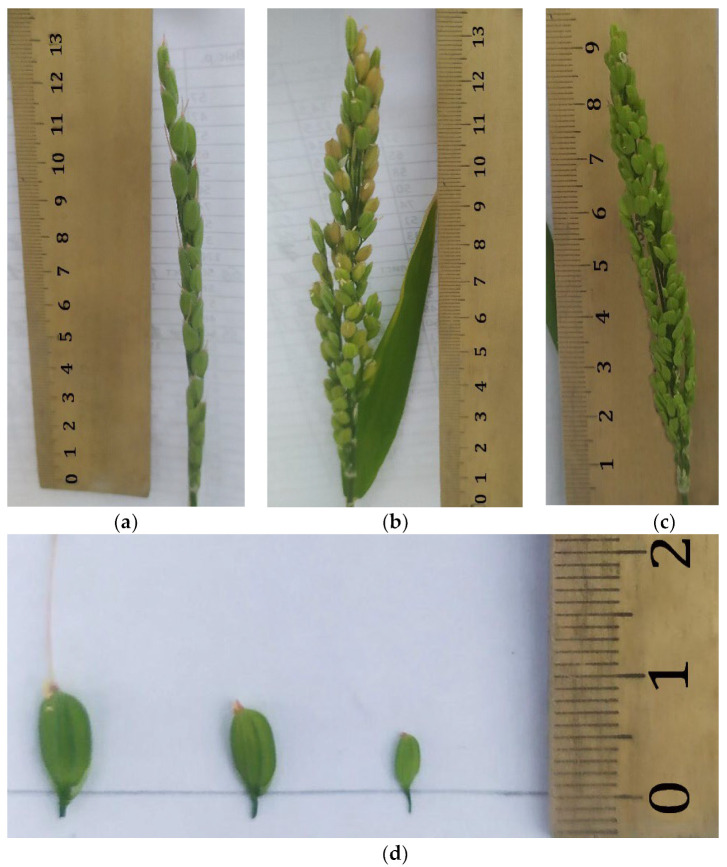
Morphological features of panicles and spikelets of rice regenerating plants: (**a**)—tetraploid, (**b**)—diploid, (**c**)—haploid, (**d**)—spikelets in order: tetraploid, diploid, haploid.

The mass of 1000 grains in diploid forms ranged from 22.1 to 30.1 g. In tetraploid forms, the mass of 1000 grains ranged from 35.5 to 51.7 g. Plants of different levels of ploidy differed significantly in the number and size of spikelets (Figure 7).

The average number of spikelets on the panicle in tetraploids was 48.8 pcs., in diploids, it was 94.0 and in haploids, it was 80.9. The spikelet length in tetraploids reached 9.0 mm, in diploids, it was 7.3 and in haploids, it was 5.0.

Morphological differences between plants did not depend on the presence of flood-resistance genes (Sub1A, Sk1), since plants did not experience this stress in the greenhouse.

The largest number of regenerating plants (14) was formed in the hybrid 4641, of which 10 were diploid and formed normal seeds.

**Figure 8:**
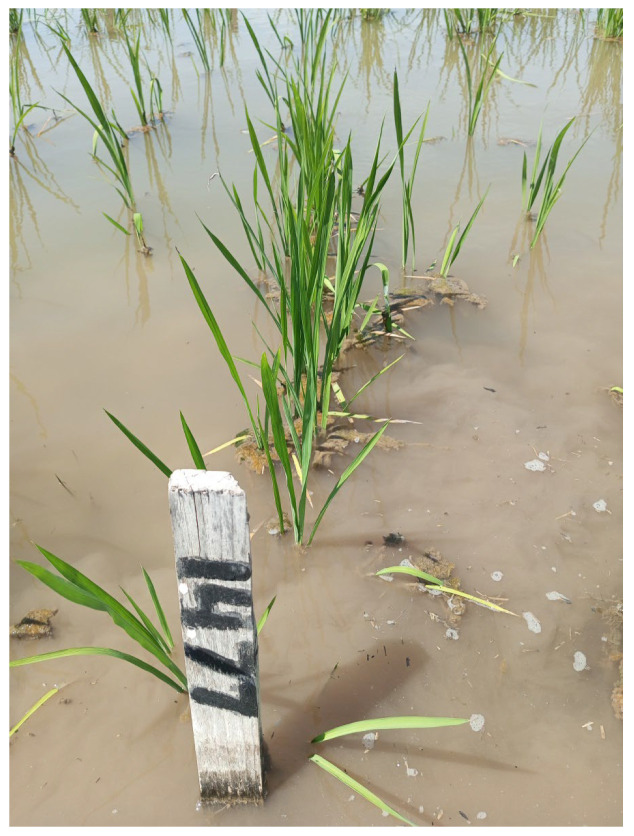
Sample 1477 from seeds of the regenerate plant 4641/1 (Inbara-3 × Contact) × (Khao Hlan On × Kubojar).

Seeds from rice regenerant plants were sown in the field. The plants that grew out of them were subjected to prolonged flooding with water to combat the hedgehog. Under these conditions, the sample 4641/1 (Inbara-3 × Contact) × (Khao Hlan On × Kubojar) carrying the Sk1 gene performed well. In the field, it was marked under the number 1477 (Figure 8).

## 4. Conclusions

The selected lines, which are characterized by good responsiveness to anther cultivation in vitro and carry the genes for resistance to prolonged flooding (Sub1A, Sk1), are used in rice breeding programs using biotechnology. On the basis of the obtained androgenic plants, dihaploid lines were formed, which will subsequently be included in breeding work to create rice varieties with economically valuable and adaptive traits.

## Figures and Tables

**Table 1:** Varieties—donors of resistance to prolonged flooding.

Variety	Gene	Country of Origin
Novator	–	Russia
Magnat	–	Russia
Contact	–	Russia
Kuboyar	–	Russia
Br-11	SUB1A	Bangladesh
CR-1009	SUB1A	India
Inbara-3	SUB1A	Indonesia
IR-64	SUB1A	Philippines
TDK-1	SUB1A	Laos
Khao Hlan On	SK1, SK2	Myanmar
Kharsu 80A	SK1, SK2	Pakistan
Ma-Zhan Red	SK1, SK2	China

**Table 2:** Composition of media for the cultivation of rice anthers.

Media Components	Induction Medium (Blaydes, 1966) (mg/L)	Regeneration Medium (Muraschige and Skoog, 1964) (mg/L)
*Macro salts*		
NH_4_NO_3_	1000	1650
KNO_3_	1000	1900
Ca(NO_3_)_2_ × 4 H_2_O	347	440
KH_2_PO_4_	300	170
MgSO_4_ × 7 H_2_O	35	370
KCl	65	–
*Micro salts*		
ZnSO_4_ × 7 H_2_O	1.5	8.6
H_3_BO_3_	1.6	1.6
MnSO_4_ × 4 H^2^O	4.4	6.92
KI	0.8	0.83
Na_2_MoO_4_ × 2 H_2_O	–	0.25
CuSO_4_ × 5 H_2_O	–	0.025
CoCl_2_ × 6 H_2_O	–	0.025
*Iron source*		
FeSO_4_ × 7 H_2_O	27.8	27.8
Na_2_EDTA	37.2	37.2
*Vitamins*		
Nicotinic acid	0.5	0.5
Pyridoxine HCI	0.5	0.5
Thiamine HCI	0.5	0.5
*Other componentss*		
myo-Inositol	100	100
Glycine	2.0	2.0
Agar	8.0	8.0
Sucrose	30.0	20.0
*Growth regulators*		
2,4-D	2.0	–
NAA	–	1.0
Kinetin	–	5.0
рН	6.0	6.0

**Table 4:** Results of cultivation of rice anthers (2022).

№	Sample №	Plant №	Inoculated Anthers, pcs.	Number of Neoplasms, pcs.	Non-Morphogenic Callus, pcs.	Total Regenerating Plants, pcs.	Green Plants, pcs.	Albino Plants, pcs.
1	5022	1	243	4	4	0	0	0
		2	275	0	0	0	0	0
		3	92	0	0	0	0	0
2	5103	2	259	20	16	4	0	4
		4	245	2	2	0	0	0
		5	110	0	0	0	0	0
3	5007	1	214	0	0	0	0	0
		3	152	0	0	0	0	0
		4	114	0	0	0	0	0
4	5005	1	299	37	34	3	0	3
		2	86	0	0	0	0	0
		3	225	1	1	0	0	0
5	5029	3	270	0	0	0	0	0
		5	132	1	1	0	0	0
		8	304	1	1	0	0	0
		10	284	0	0	0	0	0
6	5006	1	277	0	0	0	0	0
		2	189	0	0	0	0	0
		5	120	0	0	0	0	0
7	5093	1	194	1	1	0	0	0
		3	82	0	0	0	0	0
		4	272	0	0	0	0	0
8	5019	1	251	8	5	3	0	3
		2	126	3	2	1	0	1
		3	289	1	1	0	0	0
9	5003	1	306	0	0	0	0	0
		3	258	1	1	0	0	0
10	5009	1	212	0	0	0	0	0
		2	119	84	67	17	5	12
		4	278	12	12	0	0	0
11	5010	1	183	0	0	0	0	0
		2	277	94	87	7	5	2
12	5011	1	271	0	0	0	0	0
		3	186	0	0	0	0	0
13	5008	1	86	3	3	0	0	0
		2	184	0	0	0	0	0
		3	279	21	21	0	0	0
14	5020	1	243	26	15	11	0	11
		2	47	0	0	0	0	0
		3	210	13	13	0	0	0
15	5018	1	132	1	1	0	0	0
		2	298	5	4	1	0	1
		3	297	23	20	3	0	3
16	4565	2	82	3	3	0	0	0
		3	195	85	82	3	2	1
		5	245	46	43	3	0	3
17	4773	1	47	4	4	0	0	0
		2	114	0	0	0	0	0
		3	59	1	1	0	0	0
18	5016	2	339	1	1	0	0	0
		3	120	0	0	0	0	0
		4	216	0	0	0	0	0
19	4758	1	209	4	2	2	0	2
20	5021	1	248	62	37	25	0	25
		2	112	3	1	2	0	2
		3	140	0	0	0	0	0
21	4641	1	193	5	4	1	0	1
		2	194	69	48	21	18	3
22	5017	1	51	4	2	2	0	2
		2	255	2	1	1	0	1
		3	195	42	32	10	0	10
23	4526	1	80	10	3	7	0	7
24	4688	1	85	12	9	3	0	3
		2	117	0	0	0	0	0
		3	57	0	0	0	0	0
25	4617	1	83	1	1	0	0	0
26	4585	1	104	0	0	0	0	0
		2	94	0	0	0	0	0
Sum		69	12,604	716	586	130	30	100
Average		2.5	185.35	10.53	8.6	1.91	0.44	1.47
Minimum		1	47	0	0	0	0	0
Maximum		4	339	94	87	25	18	25

**Table 5:** Results of assessment of the effectiveness of anther culture of isolated rice samples (2022).

Sample №	Plant №	Number of Neoplasms/ per 100 Anthers	The Number of All Regenerants/ per 100 Anthers	Number of Green Regenerants/ per 100 Anthers	The Number of All Regenerants/ per 100 Neoplasms
5009	2	70.6 *	14.3 *	4.2	20.2
5010	2	33.9	2.5	1.8	7.5
4565	3	43.6	1.5	1.0	3.5
4641	2	35.6	10.8	9.3 *	30.4 *
Average value		45.9	7.3	4.1	15.4
Standard deviation		17.0	6.3	3.7	12.3

Note: * is a significant difference from the average value. 5009 (Inbara-3 × Novator) × Contact (gene Sub1A). 5010 (Inbara-3 × Novator) × Contact (gene Sub1A). 4565 IR-64 × Magnate (gene Sub1A). 4641 (Inbara-3 × Contact) × (Khao Hlan On × Kubojar) (genes Sub1A and SK1).

**Table 6:** Content of nuclear DNA in the population of rice regenerants obtained from second-generation hybrids in anther culture in vitro.

Index	Haploids	Diploids	Tetraploids
Number of plants, pcs.	9	11	5
DNA content, pg:			
M	0.901	1.880	3.762
±SEM	0.012	0.023	0.048
min	0.790	1.654	3.590
max	1.112	2.015	3.960
Cv, %	8.3	9.6	10.0

**Table 7:** Morphological features of rice regenerating plants.

Hybrid Number *	Plant No.	Ploidy	Sk1 Gene	Sub1A Gene	Plant Height, cm	Panicle Length, cm	Number of Panicles on a Plant, pcs	Number of Spikelets on a Panicle, pcs	Number of Grains on a Panicle, pcs	Weight of 1000 Grains, g
4565	1	1			51	11	20	128	0	0.0
4565	2	1		Sub1A	41	14	12	75	0	0.0
4641	1	2	Sk1		57	14.5	15	96	39	25.0
4641	2	2			60	13.5	9	116	52	22.1
4641	3	2	Sk1		47	12.5	10	109	60	24.8
4641	4	4			58	15.5	8	55	4	38.3
4641	5	2	Sk1	Sub1A	57	14	10	114	89	30.1
4641	6	4	Sk1		57	18	6	40	2	44.8
4641	7	2		Sub1A	65	16.5	8	126	104	28.7
4641	8	2	Sk1	Sub1A	58	15.5	8	50	32	24.2
4641	9	4	Sk1		48	13	11	36	4	47.2
4641	10	2		Sub1A	50	14	11	101	63	25.6
4641	11	2	Sk1		74	17	12	127	98	30.1
4641	12	2	Sk1	Sub1A	51	14	12	43	25	27.6
4641	13	4	Sk1	Sub1A	51	15.5	7	49	2	35.5
4641	14	4	Sk1		55	15.5	3	64	4	51.7
5009	1	1	Sk1		53	14.5	10	98	0	0.0
5009	2	2	Sk1	Sub1A	33	10	7	51	18	28.9
5009	3	1			62	13.5	40	40	0	0.0
5010	1	1		Sub1A	26	5.5	3	96	0	0.0
5010	2	1	Sk1	Sub1A	31	7.5	3	82	0	0.0
5010	3	1	Sk1	Sub1A	39	5	4	48	0	0.0
5010	4	1	Sk1		30	4	5	44	0	0.0
5010	5	1	Sk1	Sub1A	44	13	10	117	0	0.0
5010	6	2			57	14	7	101	3	20.0

* Note: Примечание: 4565 IR-64 × Magnate, 4641 (Inbara-3 × Contact) × (Khao Hlan On × Kubojar), 5009 (Inbara-3 × Novator) × Contact, 5010 (Inbara-3 × Novator) × Contact.

## Data Availability

Not applicable.
